# Comparison of rapid laboratory tests for failure of passive transfer in the bovine

**DOI:** 10.1186/s13620-015-0047-0

**Published:** 2015-08-25

**Authors:** Ian Hogan, Michael Doherty, John Fagan, Emer Kennedy, Muireann Conneely, Paula Brady, Clare Ryan, Ingrid Lorenz

**Affiliations:** Department of Agriculture Food and the Marine, Regional Veterinary Laboratory, Knockalisheen, Limerick, Ireland; Veterinary Clinical Studies, School of Veterinary Medicine, UCD, Belfield, Dublin 4 Ireland; Regional Veterinary Laboratory, Coosan, Athlone, CO. Westmeath Ireland; Animal & Grassland Research and Innovation Centre, Teagasc, Moorepark, Fermoy, Co Cork Ireland; Herd Health and Animal Husbandry, UCD School of Veterinary Medicine, Belfield, Dublin 4 Ireland

**Keywords:** Failure of passive transfer, Calves, Laboratory tests, Disadvantages in practicality

## Abstract

**Background:**

Failure of passive transfer of maternal immunity via colostrum can occur in the bovine, and a number of blood tests have been developed to test calves for this failure. It is not clear which test is most suitable for this purpose. The objective was to examine the most commonly used tests for failure of passive transfer and to decide which is most suitable for routine laboratory use. 126 serum samples were taken from calves of dairy cows after birth but prior to colostrum feeding, and at 48 h of age. Five different tests were compared against radial immunodiffusion which is considered the appropriate reference method. These tests were serum gamma-glutamyltransferase levels, serum protein levels, serum globulin levels, an enzyme linked immunosorbent assay and the zinc sulphate turbidity test.

**Results:**

The tests examined displayed high sensitivity but widely varying specificity. Examination of the use of different cut-off points allowed some improvement in specificity at the expense of sensitivity, but the tests which had performed best at the original cut-off points still displayed the best performance. Gamma-glutamyltransferase levels as a measure of colostrum absorption returned, in this study, the best balance between sensitivity and specificity. The ELISA used in this study and serum globulin levels displayed performance similar to the gamma-glutamyltransferase levels. Serum total protein was less successful than others examined at providing both sensitivity and specificity but may, when performed via refractometer, be useful for on-farm testing. As currently performed the poor sensitivity for which the zinc sulphate turbidity test is most often criticized is evident. Modification of the cut-off point to increase specificity is less successful at balancing these parameters than the ELISA, gamma-glutamyltransferase levels, and globulin levels.

**Conclusions:**

Gamma-glutamyltransferase levels, ELISA testing and circulating globulin levels performed best in detecting failure of passive transfer in serum samples, although all three had some practical considerations.

## Background

Transfer of immunity from the dam to the offspring in the bovine depends on the absorption of immunoglobulin (Ig) from colostrum consumed by the calf after birth [1]. This transfer of Ig is termed passive transfer, and failure of passive transfer (FPT) is associated in the neonate with the development of disease [2–5]. Immunoglobulins are transferred from the cow’s circulation into the colostrum [6] from where, following ingestion, they are absorbed into the calf’s bloodstream through the intestine [7]. Adequate and timely ingestion of colostrum is crucial to confer immunity to the newborn calf and reduce morbidity and mortality [8]. In addition calves cannot benefit from immunization of the dam without adequate transfer of colostral immunity [9, 10].

Failure of passive transfer may be a result of colostrum feeding practices and other herd production and management factors [11]. It will increase mortality up to 10 weeks of age, and 39 % of observed mortality in dairy calves has been attributed to it [12, 13]. Failure of passive transfer is a problem in Ireland, with only 34 % of samples submitted to Department of Agriculture laboratories returning values consistent with adequate transfer of immunity [14]. Testing of blood samples from neonatal calves to determine if FPT has occurred can form an important part of investigations into calf health problems [15].

Radial immunodiffusion (RID) measures Ig concentration directly [16, 17] and has been described as the best available test to determine Ig levels, however it is time-consuming, technically demanding and expensive, therefore it is not used for routine testing in diagnostic laboratories [18, 19]. Immunoassays, including enzyme linked immunosorbent assay (ELISA) tests are now commercially available; these also directly measure the Ig concentration in the serum.

A number of tests have been devised which attempt to measure Ig levels indirectly. One type of test involves the precipitation of Ig using solutions of metal salts. This causes turbidity, which is assessed either visually or by colourimeter, giving a result in units of turbidity. Examples include tests using sodium sulphite [19] (SST) and zinc sulphate (ZST) [20].

The protein in serum from clotted blood samples consists of albumin and globulin, the latter made up of Ig and non-immune globulin. Serum total protein levels (STP) are considered to rise as Ig and other proteins are absorbed from colostrum and may be used as an indicator of Ig absorption. On-farm measurement of STP is possible by refractometry, as the specific gravity of serum is indicative of STP concentration [21–23]. Most authors suggest that this test is not suitable for use in sick, dehydrated or moribund calves [15, 23, 24]. Concern has also been expressed about possible variations in albumin levels [25].

The level of globulin in a sample should similarly have risen when Ig and other globulins were absorbed from colostrum into the neonate’s circulation, in a similar manner to STP but without any variation in albumin levels. It has been suggested that the non-immunoglobulin component of serum globulin concentration is approximately 1.0 to 1.5 g/dL, so the remaining serum globulin represents immunoglobulin [15]. Globulin levels as an indicator of FPT are not as widely discussed in the literature as STP [21–23]. This may be because STP can easily be determined on-farm via a refractometer, while globulin levels cannot. In a laboratory context, however, analysis of globulin levels in a sample is no more difficult than STP levels alone.

The alternative to direct and indirect measurement of Ig levels is the assessment of the levels in the blood of other components of colostrum which are similarly absorbed. Gamma-glutamyltransferase (GGT) is present in colostrum and its concentration in neonatal serum rises dramatically in line with absorption of colostrum constituents. A number of studies [24, 26, 27] describe how elevated blood GGT levels in the first few days post-partum are indicative of absorption from colostrum. The period where this test could be used was estimated to be less than eight days of age in beef calves [28] and up to ten days of age in dairy calves [29].

Other tests which have been proposed to estimate Ig concentrations include a gluteraldehyde coagulation test [30] and a commercially available latex agglutination test [31]. Serum electrophoresis can also be used [25].

The current study was conceived in order to determine which of the various tests was most appropriate for routine laboratory use. Samples from calves before and after colostrum consumption were subjected to several of the above described tests, including GGT, STP, globulin, ZST, and a commercially available ELISA. A commercially available RID was used as a reference method to compare the performance of the tests.

## Methods

One hundred and twenty six serum samples for comparison of tests were collected from Friesian calves at the Teagasc, Moorepark Animal & Grassland Research and Innovation Centre, Fermoy, Co. Cork, Ireland. These calves were part of a study involving feeding colostrum at different volumes and varying the subsequent number of transition milk feeds, and the blood sampling had been undertaken in the course of this [32]. Calves were fed different volumes of colostrum (7, 8.5, or 10 % of birth body weight) within 2 h of birth, and given either 2 or 4 additional feedings of transition milk. All calving events were observed and attended by trained and competent farm personnel, the calf was removed from the dam before it became ambulatory, and the calf was fed the volume of colostrum dictated by its experimental treatment group via stomach tube [32].

A 6 ml blood sample was taken from the jugular vein of each calf into a plain serum tube (*BD Vacutainer, Lagenbach)* within 1 h of birth, prior to feeding colostrum (0 h). Blood samples were taken again from each calf 48 h after the initial feed of colostrum. Blood samples were refrigerated for 24 h before serum was separated by centrifugation and frozen at -20 °C before IgG concentration determination.

Samples were tested using an ELISA for immunoglobulin G (Bovine IgG Elisa Kit Cat. No. 8010, Alpha Diagnostic International) at Teagasc, Moorepark Animal & Grassland Research and Innovation Centre and refrozen. The frozen samples were then transported to Limerick Regional Veterinary Laboratory (RVL) where they were maintained at -20 °C until thawing for further testing.

Samples were tested by ZST using the standard operating procedure in place at Limerick RVL. This was as described in McEwan [20] with the modification that the concentration of the zinc sulphate solution used was 250 mg/L rather than 208 mg/L, as employed by Hudgens [33].

Testing for GGT, STP and albumin was carried out using an Rx Daytona autoanalyser. Gamma-glutamyltransferase levels were evaluated by a colourimetric method where the L-γ-glutamyl-3-carboxy-4-nitroanilide is converted in the presence of glycylglycine by GGT to 5-amino-2-nitro-benzoate which absorbs at 405 nm [34]. Total protein levels were determined by formation of a coloured complex between protein and cupric ions in an alkaline medium [35]. Albumin levels were determined by quantitative binding to the indicator 3,3′,5,5′-tetrabromo-m cresol sulphonphthalein (bromocresol green) [36].

Globulin levels were determined by subtracting albumin levels from STP. After a thorough literature review, an appropriate cut-off point for globulin levels (below which FPT might be deemed to have occurred) was not found to be established by experiment. However some authors have speculated that since the non-immunoglobulin component of serum globulin has been suggested as between 1 and 1.5 g/dL [15], a level of serum globulin-consisting of both immunoglobulin and the non-immune component- under 20 or 25 mg/ml respectively would indicate an immunoglobulin concentration less than 10 mg/ml. An appropriate cut-off point would be likely to lie within this range.

To provide a gold standard 0 h and 48 h samples were tested with a commercially available RID kit [16] (Triple J farms Bovine IgG kit product #728411). Immunoglobulin G levels are considered a good indicator of total immunoglobulin levels [37]. 1000 mg/dL, or 10 mg/ml, is the threshold below which passive transfer is considered inadequate [1, 23, 38–40].

Statistical analysis was carried out using the Epitools epidemiological calculators provided by AusVet Animal Health Services and Australian Biosecurity Cooperative Research Centre for Emerging Infectious Disease [41]. The characteristics in question were the sensitivity (*Se*) or the ability of a test to detect animal with FPT correctly, and its specificity (*Sp*) or the ability to give the correct answer if the animal in question is not suffering from FPT [42]. Results were first analyzed to determine *Se, Sp* and 95 % confidence intervals thereof, for each test, using the cut-off points described in the literature. The cut-off points used were 20 units in the case of the ZST test [20], 52 mg/ml in the case of STP [38], 20 mg/ml in the case of globulin [15] and 100 IU/L for GGT levels [29]. As a direct test the ELISA uses an identical cut-off point to the reference method, 10mg/ml. It is the nature of tests returning a numerical value that the cut-off point dividing positive from negative results can be adjusted to increase the sensitivity at the expense of specificity, or vice-versa, depending on the comparative cost of a false positive or false negative result [42]. Receiver operating characteristic curves (ROC curves) were used to determine the various cut-off points which return maximum Se, Sp, and maximum number of animals classified correctly [42]. The area under the curve (AUC) of the ROC curve is a measure of the overall ability of the test to discriminate affected from unaffected animals [42]. In very high value animals maximizing sensitivity may be desired due to the greater financial cost of mortalities that might result from FPT [33]. Assuming equal costs of false negative and false positive test results the optimal output is that with *Se* + *Sp* at a maximum, this occurs where the curve gets closest to the top left corner of the ROC curve. Sensitivity, specificity and confidence intervals could then be examined at the “optimum” cut-off point as well as at the “recommended” cut-off point.

## Results and discussion

A range of studies have been carried out comparing the performance of tests of FPT. GGT levels [23, 24, 29], STP levels [19, 21–25, 38, 39] and the ZST test [18, 21, 22, 25, 39, 43, 44] have frequently been examined, as occasionally have commercially available immunoassays [45]. STP has demonstrated practical advantages, while GGT has also been found to perform well by many studies. ZST testing has generally displayed high *Se* but very poor *Sp*, although many of the tests where *Sp* has been poorest have used visual reading rather than photometric. These findings have been reported in reviews and articles on calf health [1, 15, 46]. Although globulin levels have been suggested as a test for FPT [15] an examination of the performance has not been found in the literature. The current study attempts to examine the performance of globulin levels and ELISA as tests for FPT in comparison to tests which have received closer examination.

**Fig. 1:**
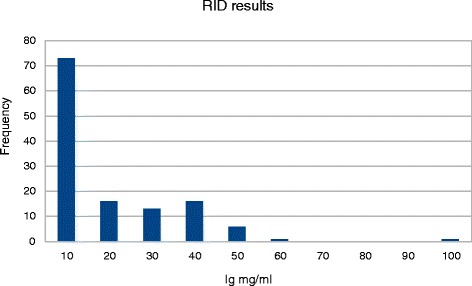
The distribution of Immunnoglobulin G results from 126 samples from dairy calves,determined by radial immunodiffusion testing [63]. Levels less than 10 mg/ml (1000 mg/dL) are considered indicative of failure of passive transfer [1, 23, 38–40]

One hundred and twenty six samples were examined by all tests. Samples from pre-colostral calves were used as a source of serum with low circulating immunoglobulins. In 73 of these IgG levels were determined by RID to be less than 10 mg/ml, and in 53 samples IgG levels determined to be greater than 10 mg/ml. Of the samples tested, 58 % had IgG concentrations below 1000 mg/dL, or 10 mg/ml, so prevalence (*P*) of samples where adequate transfer did not take place was found to be 0.58. The distribution of IgG level results obtained by RID is depicted in Fig. 1.

The performance of the tests involved at the cut-off points recommended in the literature was examined and can be seen in Table 1. Positive and negative predictive values have been calculated based on the reported prevalence of FPT in Irish calves [14]. It can be seen that at the cut-off points recommended in the literature that all tests examined returned high sensitivity, none lower than 97 %, in fact the 95 % confidence limits for sensitivity of all five tests overlap. However the specificity of the various tests is quite variable, as low as 30 % in the case of the ZST test and 57 % for STP. Globulin levels return specificity of 85 % at the recommended cut-off point while for GGT and ELISA it is over 98 %, but the confidence intervals of these three specificities overlap. A kappa (or Cohen’s kappa) statistic may be used to measure the level of agreement, beyond chance, between tests [42]. The kappa statistics between the tests in the current study is presented in Table 2, along with the McNemar’s *χ*^2^ statistic comparing the *Se* and *Sp* of the tests. Unsurprisingly given the uniform high sensitivity, it may be seen that agreement is highest between tests with the highest specificity, namely GGT, ELISA and globulin levels.

**Table 1:** Performance of various tests in identifying failure of passive transfer, compared to radial immunodiffusion as a reference method, at the cut-off points recommended in the literature. The uniformly high sensitivity, and the widely varying specificity, may be noted. Positive and negative predictive values have been calculated based on the reported prevalence of FPT in Irish calves [14]

Test	Recommended cut-off point	Sensitivity(95 % CI)	Specificity (95 % CI)	% of animals classified correctly	Positive predictive value	Negative predictive value
GGT	100 IU/L [29]	0.9726 (0.9045-0.9967)	0.9811 (0.8993-0.9995)	98 %	0.9901	0.9486
ELISA	10 mg/ml [39]	0.9726 (0.9045-0.9967)	0.9811 (0.8993-0.9995)	98 %	0.9901	0.9486
Globulin	20 mg/ml [15]	1.0000 (0.9507-1.0000)	0.8491 (0.7241-0.9325)	94 %	0.9279	1.0000
STP	52 mg/ml [38]	1.0000 (0.9507-1.0000)	0.5660 (0.4228-0.7016)	82 %	0.8173	1.0000
ZST	20 units [20]	0.9863 (0.9260-0.9997)	0.3019 (0.1834-0.4434)	70 %	0.7328	0.9190

**Table 2:** The degree of agreement (kappa) between various tests when carried out using the cut-off points recommended in the literature. Also shown are the McNemar’s *χ*2 statistics comparing the sensitivity and specificity of the tests. McNemar’s test did not return a result when comparing the *Sp* of GGT levels and the ELISA as the tests returned the exact same results in unaffected animals –the specificity was identical. The test did not return a result when comparing the *Se* of Globulin and STP levels for the same reason- the sensitivity of these tests was identical

Recommended cut-off	Kappa	McNemar’s *χ*2 for *Se*	McNemar’s *χ*2 for Sp
GGT v ELISA	0.9676	0	No result
GGT v Globulin	0.8851	0.5	5.1
ELISA v Globulin	0.8511	0.5	5.1
Globulin v STP	0.7200	No result	13
GGT v STP	0.5882	0.5	20
ELISA v STP	0.5882	0.5	20
GGT v ZST	0.3089	0	34
ELISA v ZST	0.3089	0	34
Globulin v ZST	0.2378	0	21
STP v ZST	0.1518	0	5.3

**Fig. 2:**
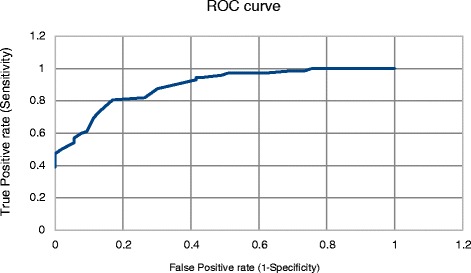
Example of a receiver operating characteristic (ROC) curve, describing the performance of the zinc sulphate turbidity test [42]. Similar curves were created for all tests under examination

An ROC curve is used to assess the discriminating ability of each test over a range of cut-off points. An example of such a curve can be seen in Fig. 2. The AUC results of the ROC curves are outlined in Table 3. It can be seen that this measure is highest for the GGT level, followed closely by globulin and ELISA. Using a published arbitrary guideline [47] the GGT, ELISA and globulin could be described as highly accurate (0.9 < AUC < 1) while the STP and ZST were moderately accurate (0.7 < AUC ≤ 0.9). As the cut-off point of the test is changed to maximize *Se,* the *Sp* is reduced, and vice versa, and it may be seen in Table 3 that either 100 % *Se* or *Sp* will require significant compromise in the other measure. The ELISA is best able to maintain good *Se* as *Sp* is maximized, keeping a *Se* over 97 %, while the globulin level can maintain *Sp* of almost 85 % at maximum *Se.* The tests which displayed the poorest specificity at the recommended cut-off points, the ZST test and the STP level, are tests which are most unable to compromise *Se* and *Sp.*

**Table 3:** Cut-points determined, by use of receiver operating characteristic (ROC) curve, to maximize either the sensitivity or the specificity of each test [42]. The area under the curve is a measure of the overall ability of the test to discriminate affected from unaffected animals [42]

Test	Area under curve (AUC)	Cut-point (Target *Se*)	Se	Sp	Cut-point (Target *Sp*)	Se	Sp
GGT	0.996	3449.3 IU/L	100.00 %	0.00 %	4.6 IU/L	80.80 %	100.00 %
ELISA	0.989	21.9 mg/ml	100.00 %	73.60 %	6.4 mg/ml	97.30 %	100.00 %
Globulin	0.98	19.11 mg/ml	100.00 %	84.90 %	12.65 mg/ml	86.30 %	100.00 %
STP	0.894	48.9 mg/ml	100.00 %	69.80 %	25.4 mg/ml	21.90 %	100.00 %
ZST	0.875	21 units	100.00 %	24.50 %	1 unit	47.90 %	100.00 %

The performance of the tests when cut-off points returning maximum *Se* + *Sp* are used can be seen in Table 4. The tests that performed best at recommended cut-off points- GGT, ELISA and globulin-are also those which have optimum cut-off points closest to those recommended. As a direct test of IgG levels the ELISA might be expected to have an optimum cut-off point equal to that of the reference method, but it displayed better performance at 8mg/ml, due to a slightly higher specificity. The level of agreement between the tests when read at these cut-off points is expressed as a kappa statistic in Table 5, along with the McNemar’s *χ*^2^ statistic comparing the *Se* and *Sp* of the tests. Agreement between tests is improved across the board but is still greatest between ELISA, GGT levels and globulin levels.

**Table 4:** Performance of the tests at cut-off points deemed to return the maximum sum of sensitivity and specificity, as determined by receiver operating characteristic curves [42]. Positive and negative predictive values have been calculated based on the reported prevalence of FPT in Irish calves [14]

Test	Cut-off to maximise correct classification	Sensitivity(95 % CI)	Specificity (95 % CI)	% of animals classified correctly	Positive predictive value	Negative predictive value
GGT	100 IU/L	0.9726(0.9045–0.9967)	0.9811(0.8993–0.9995)	98 %	0.9901	0.9486
ELISA	8 mg/ml	0.9726(0.9045–0.9967)	1.0000(0.9328–1.0000)	98 %	1.0000	0.9758
Globulin	16 mg/ml	0.9863(0.9260–0.9997)	0.8868(0.7697–0.9573)	94 %	0.9442	0.9709
STP	45 mg/ml	0.9726(0.9045–0.9967)	0.7358(0.5967–0.8474)	87 %	0.8772	0.9326
ZST	11 units	0.8082(0.6992–0.8910)	0.8302(0.7020–0.9193)	82 %	0.9023	0.6904

**Table 5:** The degree of agreement (kappa) between various tests when carried out at cut-off points deemed to return the maximum sum of sensitivity and specificity, as determined by receiver operating characteristic curves [42]. Also shown are the McNemar’s *χ*2 statistics comparing the sensitivity and specificity of the tests. McNemar’s test did not return a result when comparing the *Sp* of GGT levels and the ELISA as the tests returned the exact same results in unaffected animals –the specificity was identical

Optimum cut-off	Kappa	McNemar’s *χ*2 for *Se*	McNemar’s *χ*2 for Sp
GGT v ELISA	0.9676	0	No result
GGT v Globulin	0.9014	0	3.2
ELISA v Globulin	0.8685	0	3.2
Globulin v STP	0.8096	0	6.1
GGT v STP	0.7160	0	11
ELISA v STP	0.7160	0	11
ELISA v ZST	0.6957	7.7	6.1
GGT v ZST	0.6688	10	6.1
Globulin v ZST	0.5917	11	0.3077
STP v ZST	0.4613	7.6	0.9412

The tests which performed best in this study, maximizing both *Se* and *Sp,* were the ELISA, the measurement of GGT levels, and the measurement of globulin levels. Aside from the efficacy of these tests at returning correct results, practical considerations may influence which test is most appropriate for use in particular circumstances.

Testing of blood samples from neonatal calves to determine if FPT has occurred can be carried out on an individual or herd basis. If performed on samples from individual calves it may be difficult to determine whether inadequate immunity or excessive challenge is the greater factor in causing disease [15]. In very high value animals where measures such as Ig transfer via plasma transfusion might be considered, a test of high sensitivity may be indicated [33].

Of much more use may be the monitoring of herd colostrum management programs; a minimum of 12 samples from clinically normal calves is suggested [1]. Such monitoring may also form part of an investigation into an outbreak of disease in the herd [15], and enable a decision into whether inadequate passive transfer is contributing to an excess mortality rate [48]. The proportion of calves in a herd suffering from FPT is of more significance than the average serum Ig concentration [11]. Recommendations to improve colostrum feeding management can be made to herd-owners on the basis of these results, if appropriate.

### GGT

GGT is present in colostrum in much greater concentrations than in milk or in the maternal serum [49]. GGT concentrations in neonatal serum rise dramatically as it and other enzymes are absorbed from colostrum, and it may be used as an indicator of colostrum intake [49]. Despite wide inter-individual variations of GGT concentration in the cow’s colostrum studies have found the GGT level in calf serum to a good qualitative indicator of colostrum intake [24, 26] and as having 80 % *Se* and 97% *Sp* in detecting calves with IgG levels <800 mg/ml [24]. Studies found GGT to correctly classify the highest percentage- compared to ZST, SST and refractometry (STP)-of clinically ill calves with regard to their passive transfer status [23]. Other works have preferred IgG levels as an indicator of colostrum intake [27]. Different cut-off points have been recommended for GGT levels as an indicator of FPT, and the cut-off point chosen determines the age of calf in which this test can be used. Suggested cut-off points include 200 IU/L in healthy 1 − 7 day old calves [24], and in another paper (based on a regression model against the age of the calf), 200 IU/L at day one, 100 IU/L at day four, 75 IU/L at one week and 50 IU/L in the second week of life [29]. The test was described as “moderately accurate” and a “reasonable choice” for diagnosis of FPT in individual lambs [50]. The period of life when this test could be used was estimated as the first two [27], eight [28] or ten days [29]. One study suggested this test be restricted to calves under eight days of age, and that it has no advantages over refractometry in beef calves, theorizing that favourable results in previous studies involving dairy calves had been due to the higher prevalence of FPT in dairy herds [28]. A review discussing these contradictory findings advised that the use of GGT levels for this purpose should be discouraged [46].

GGT levels as a measure of colostrum absorption returned in this study the best balance between sensitivity and specificity. A cut-off point of 100 IU/L was used as all calves sampled were less than four days of age [29], and this proved to be the optimum cut-off point when examined using a ROC curve. When the GGT level is determined using an automatic analyser, as in this study, it is cheap, conveniently automated, and quick. It has also flexibility regarding the number of samples to be tested; anything from a single sample to relatively large numbers can be accommodated in one run of the machine.

### ELISA

In previous studies immunoassays, including commercially available immunoassays, have performed well in detecting calves with FPT [45]. The ELISA is a direct measure of circulating IgG levels, which have been claimed to be a superior indicator of colostrum intake as it may be used over a longer time period of the animal’s life than GGT [27]. However other studies suggest the GGT level can be used in slightly older calves [28, 29] and there are doubts over the use of IgG levels after the first week of life. Endogenous production of IgG_1_ has been shown to occur in calves between the ages of 36 h and three weeks of age at the rate of about 1g of IgG_1_ per day [51]. Another study demonstrated detectable Ig (largely IgM) produced by colostrum deprived calves after seven days, while calves that derived high concentrations of Ig from colostrum did not produce endogenous Ig until four weeks of age [52]. In another study a high correlation of 0.97 was noted between circulating Ig levels in the 1^st^ and 2^nd^ week of life of each calf; this was despite the tendency to move to a central concentration, attributed to endogenous Ig production in calves with low initial Ig values and dropping Ig levels in calves with initial high levels [48]. It was concluded that the correlation was sufficiently rigid to make 2^nd^ week concentrations highly predictive of those in the first week. However sampling was still deemed preferable in week one of life, an important reason being the substantial portion of total mortality risk that occurs during the 1^st^ week. Since sampling is best used to examine herd colostrum management it is recommended that calves should be sampled between 24 h and seven days of age [1].

The ELISA used in the present study performed similarly to the measurement of GGT levels. It should be noted that an extra freeze-thaw cycle occurred between carrying out of the ELISA test on the samples and the other tests, including RID testing. Repeated freeze-thaw cycles will reduce the levels of serum proteins, and Ig has been shown to degrade when colostrum is frozen and thawed [53]. The possible effect on the current study would be a potential higher concentration of IgG in the samples at the time of ELISA testing compared to the time of RID testing, which would have the effect of lowering the apparent *Se* of the ELISA and raising it’s apparent *Sp*. Despite this risk both the *Se* and *Sp* of the ELISA were high, at 97 and 98 % respectively.

The only possible drawbacks are the cost of the test, and the fact that the ELISA is a more technically demanding procedure. This ELISA is carried out by kits containing 12 wells, if a lesser number of samples needs to be tested this leads to wastage and increased “per-unit” cost. A scenario where the ELISA might be less ideal is one where low numbers of samples require testing quickly, and cannot wait until batches of 12 are assembled.

### Globulin

Serum globulin levels displayed a performance in detecting FPT that approached those of the ELISA and GGT levels. In the present study GGT, globulin and STP were determined by autoanalyser which has many practical advantages. It is cheap, costing only several cents per sample. It is quick, single samples or small batches returning a result in 15 min. It is flexible with regard to the number of samples; the model used in the present study can test between one and forty samples at a time. The cut-off point for globulin levels where optimum sensitivity and specificity were attained was much lower than had been anticipated previously [15]. Further work might be required to refine the cut-off point established here for this test, and to examine if the caveats expressed regarding the unsuitability of the STP test for samples from sick or dehydrated calves apply to this test also.

The confidence intervals for both *Se* and *Sp* of GGT, ELISA and globulin overlap both at the cut-off points recommended in the literature and at the optimum cut-off points determined by ROC curve. Comparing any two of these tests using kappa–again at both recommended and optimum cut-off points-returns a kappa statistic in the range 0.81 − 1.00, which has been described as almost perfect agreement [54]. There is thus no significant difference in the performance of these three tests, and test selection can be made on a basis of practicality and availability.

### Serum total protein concentration

The serum total protein (STP) concentration of samples from calves can be indicative of immunoglobulin concentrations, as ingestion of immunoglobulins will raise STP levels in the blood. Serum protein levels are preferred to plasma protein levels as the latter gives a poorer correlation with immunoglobulin levels [22].

A number of cut off points for STP have been suggested, most authors suggesting between 5.0 or 5.5 g/dL of serum total protein, depending on whether sensitivity or specificity is prioritized [23, 38, 39]. An optimum point of 5.2 g/dL has been mentioned [38, 39]. A lower cut-off point of 4.2 g/dL has been proposed but this was to identify IgG levels less than 800 mg/dL [24].

This test has been found to be effective at detecting FPT in a number of studies [21–23, 39]. Others have expressed concern that variation in the non-immune component of serum protein might lead to misclassification of the FPT status of calves [25]. An attempt to combat this by separating IgG from other proteins with caprylic acid, a short-chain fatty acid, did not improve the overall predictability of refractometry [55]. Serum total protein is recommended as an on-farm screening test of healthy calves to evaluate farm colostrum management [1, 21, 23, 38].

Most of the works cited describe this test being carried out using a refractometer, with a view to on-farm use. The practical advantages of refractometry, together with results comparable to other semi-quantitative methods, lead to the test being recommended for foals [44]. Proteins in solution cause a change in refractive index of the solution that is proportional to the concentration of the protein [56]. The performance of the refractometers themselves were not examined in this study, but previous comparison of different brands of temperature-compensating and non-temperature-compensating refractometers has shown they perform similarly in detecting FPT, although non-compensating refractometers have other limitations [38]. Results of total solids refractometry from centrifuged and non-centrifuged serum have been shown to be highly correlated. Results from digital and hand held refractometers were also highly correlated [57].

In the present study STP was determined by autoanalyser and shares the advantages that measurement of GGT and globulin results gained by using this method. The cut-off points which return the best compromise between *Se* and *Sp* are much lower than those suggested by the literature, suggesting that levels of albumin and non-immune globulin in the serum from calves in this study are much lower than the literature would have anticipated [25]. This test was less successful than others examined at providing both sensitivity and specificity. STP is not recommended for dehydrated calves or those with a protein-losing enteropathy [15, 23, 24] so might not be ideal in a laboratory where samples may arrive with an incomplete history. Some authors suggest using packed cell volume as a proxy for hydration status to improve accuracy [23].

When performed via refractometer, it is the test of choice where on-farm testing is required for screening for FPT [1, 23, 24]. The convenience of on-farm testing may allow greater numbers of samples to be tested, which might compensate for the lower *Se* and *Sp*.

### Zinc sulphate turbidity test. (ZST)

This test involves the precipitation of high molecular weight proteins out of solution causing turbidity, which is assessed via the use of a colourimeter [20]. Studies have found correlation between the ZST result and both IgG levels [22, 58, 59] and levels of total immunoglobulin [20, 22, 59, 60]. Studies have also shown a relationship between low ZST scores and poor health status of calves [2, 61, 62]. Concerns have been expressed about the effect of haemolysis of the samples, time, temperature of the reaction and by the action of CO2 on the ZnSO4 solution, and modifications have been proposed to reduce errors due to haemolysis [25]. These concerns have also been raised about the use of the test in foals [44]. A simplified ZST test has also been developed for “pen-side “use, which eliminates the use of a control tube and instead uses the legibility, or illegibility of newsprint through the solution as criteria to assess turbidity [39]. This simplified test was found to be 100 % sensitive but only marginally specific (52 %) and it was determined that the endpoint was set too high. Further testing found this simplified, qualitative version of this test to be sensitive (93 %) but not specific (33 %) [23]. Gradually increasing the concentration of the zinc sulphate test solution in the qualitative ZST test it was shown that specificity would improve and sensitivity would decline [33]. Various solution strengths between 250 and 400 mg/L are suggested depending on whether specificity or sensitivity is considered more important. A concentration strength of 250 mg/L was used in the current study in the only modification to the original test [20].

The ZST test is simple, cheap and flexible, requiring some glassware and a few reagents and returns a result in an hour. However as currently performed the poor sensitivity for which the ZST test is most often criticized is evident. Modification of the cut-off point to increase specificity, as expected, leads to reduced sensitivity, however ZST test is less successful at balancing these parameters than the ELISA, GGT level, and Globulin level.

## Conclusions

The tests examined displayed high sensitivity but widely varying specificity. Examination of the use of different cut-off points allowed some improvement in specificity at the expense of sensitivity but the tests which had performed best at the original cut-off points still displayed the best performance. GGT levels, the ELISA and globulin levels returned, in this study, the best balance between sensitivity and specificity, and there was no significant difference in the performance of these tests. STP was less successful than other tests examined at providing both sensitivity and specificity but may, when performed via refractometer, be useful for on-farm testing. As currently performed the poor specificity for which the ZST test is most often criticized is evident. Modification of the cut-off point to increase specificity is less successful at balancing these parameters than it is for the ELISA, GGT level, and Globulin level.

## Abbreviations

Ig: Immunoglobulin
FPT: Failure of passive transfer
RID: Radial immunodiffusion
ELISA: Enzyme linked immunosorbent assay
SST: Sodium sulphite turbidity
ZST test: Zinc sulphate turbidity test
STP: Serum total protein
GGT: Gammaglutamyl transferase
IgG: Immunoglobulin G
*Se*: Sensitivity
*Sp*: Specificity
ROC curves: Receiver operating characteristic curves
AUC: Area under the curve
